# A comparison of the use of propofol alone and propofol with midazolam for pediatric magnetic resonance imaging sedation – a retrospective cohort study

**DOI:** 10.1186/s12871-017-0431-2

**Published:** 2017-10-11

**Authors:** RyungA Kang, Young Hee Shin, Nam-Su Gil, Ki Yoon Kim, Hyean Yeo, Ji Seon Jeong

**Affiliations:** 1 Department of Anesthesiology and Pain Medicine, Samsung Medical Center, Sungkyunkwan University school of Medicine, 50, Ilwon-Dong, Kangnam-Ku, Seoul, 06351 Korea; 2 Department of Anesthesiology and Pain Medicine, Kangwon National University School of Medicine, Chuncheon, Korea

**Keywords:** Magnetic resonance imaging (MRI), Midazolam, Pediatric sedation, Propofol

## Abstract

**Background:**

Pediatric MRI sedation performed by a variety of specialists such as sedationists and anesthesiologists commonly uses propofol, which has similar effects to an ideal sedative agent for maintaining deep sedation. However, when propofol is used, adverse airway events are relatively more common than when using other sedative agents. The concomitant administration of midazolam and propofol can reduce the dose of propofol needed for adequate sedation and might also reduce the frequency of airway obstruction without affecting the patient’s recovery profile.

**Methods:**

We reviewed the our hospital records of all pediatric MRI sedation patients aged 3 to 16 years who were sedated with either propofol alone or propofol with midazolam between December 2013 and June 2016.

**Results:**

Eight hundred ninety-seven pediatric MRI sedation patients were included (*n* = 897). The frequency of airway intervention was 25/356 (7.0%) in Group P and 15/541 (2.8%) in Group PM (difference in proportions: 4.2%; 95% CI: 1.4–7.6%; *p* = 0.002). The mean (SD) time to awake was longer in Group PM compared to Group P [21.2 (5.6) minutes vs. 23.0 (7.1) minutes; mean difference, 1.8 min; 95% CI, 0.9–2.9; *p* < 0.001]. The mean (SD) time to discharge was longer in Group PM compared to Group P [34.5 (6.9) minutes vs. 38.6 (9.4) minutes; mean difference, 4.0 min; 95% CI, 3.0–5.1; p < 0.001].

**Conclusions:**

The administration of a small dose of midazolam during pediatric MRI sedation using propofol can reduce the frequency of airway complications without prolonging the clinically significant recovery profile.

## Background

Magnetic resonance imaging (MRI) is a widely used diagnostic imaging tool in pediatric patients and typically takes between 30 to 60 min to perform. Therefore, cooperation of pediatric patients is essential during MRI. Often, pediatric patients need sedation because proper cooperation is difficult to obtain. Deep sedation is required to obtain high quality images in order to ensure a fixed posture and to prevent involuntary movement due to noise [1–3].

Ideal sedative agents should have rapid onset time, short recovery profile, and low potential for side effects. Pediatric MRI sedation performed by a variety of specialists such as sedationists and anesthesiologists commonly uses propofol, which has similar effects to an ideal sedative agent for maintaining deep sedation [4]. However, adverse airway events are more common than with other sedative agents [2].

Airway collapsibility is known to be proportional to the effect-site concentration of propofol [5]. In sedation using propofol, concomitant administration of midazolam can reduce the dose of propofol needed for adequate sedation [6]. Therefore, concomitant administration of midazolam can reduce the dose of propofol used and might also reduce the frequency of airway obstruction without affecting the recovery profile. The objective of this study was to compare the recovery profiles and airway-related adverse events of pediatric MRI sedation patients who received propofol alone to those who received propofol with midazolam.

## Methods

This retrospective cohort study was approved by the institutional review board of Samsung Medical Center (IRB No. SMC 2017–04-006). A search of the electronic medical record database was conducted between December 2013 and June 2016 to identify children aged 3 to 16 years who received propofol with or without midazolam for MRI sedation. Patients with tracheostomy, who were intubated, or who had laryngomalacia, a neck mass, a known airway problem, another procedure after MRI sedation, premedication, or use of other sedative agents were excluded. Also, cases that used midazolam for more than just induction of sedation during the procedure were excluded from the study. An independent investigator collected data from the electronic medical records to avoid reporting bias.

The sedation procedure used in this study was as follows. After the patient arrived in the waiting room, she/he underwent a physical examination, and MRI sedation was performed if there were no abnormalities. A 24-gauge intravenous catheter was inserted into the forearm 30 min before the MRI scan. In the MRI room, standard monitoring of vitals, including electrocardiography, non-invasive blood pressure monitoring, and pulse oximetry, was performed. The patients were divided into two groups: those who received propofol alone (Group P) and those who received propofol with midazolam (Group PM) for pediatric MRI sedation. In Group P, a 1–2 mg/kg of propofol bolus was administered to achieve deep sedation (Ramsay Sedation Scale score of 5). Patients in Group PM received 0.05 mg/kg of midazolam before administration of propofol. In both groups, if deep sedation was not achieved, an additional 1 mg/kg of propofol was given until deep sedation was achieved. During maintenance of sedation, every patient received 150 μg/kg/min of propofol, and the infusion rate was adjusted in 25 μg/kg/min increments up or down at the discretion of the anesthesiologists in order to maintain deep sedation. If involuntary movement was present, a supplemental dose of propofol (0.5–1 mg/kg) was administrated or the infusion rate was increased in 25 μg/kg/min increments. During MRI sedation, oxygen was administered at 4 L/min via a facial mask with the metal piece removed, and continuous nasal end-tidal carbon dioxide monitoring was performed. The patients were placed in the supine position with a roll under their shoulders, and noise-reduction earplugs were worn.

Standard interventions for apnea (>10 s), oxygen desaturation (SpO2 < 90%), and airway obstruction were performed, which included checking for equipment malfunctions, airway repositioning, the chin lift/jaw thrust maneuver, or bag-mask ventilation as needed. If there was no improvement, other airway devices such as an oral/nasal airway, a laryngeal mask airway (LMA), or an endotracheal tube, were applied. When the pediatric MRI sedation failed despite the use of these other airway devices, general anesthesia using inhaled anesthetics and muscle relaxants was performed. After the MRI was completed, the propofol was disconnected. The patient was then transferred to the post-anesthesia care unit, and standard monitoring was performed until the patient was discharged. All procedures for pediatric MRI sedation were performed by a pediatric anesthesiologist. The quality of MRI images was assessed by the MRI technician after each sequence; if needed, the sequence was repeated.

The primary outcomes of this study were recovery profiles (time to awake and time to discharge) and airway-related intervention ratios in pediatric MRI sedation patients. Patient demographics, MRI sedation, and recovery data, including propofol induction dose, airway intervention and sedation-related adverse events from the pediatric sedation recovery unit were also collected.

### Statistical analysis

The statistical analyses were performed using IBM SPSS 22 for Windows (SPSS Inc., Armonk, NY, USA). Continuous variables are presented as mean ± SD, and categorical variables are presented as number (%). T-test or Mann–Whitney U test was used for continuous variables, and Chi-square tests or Fisher’s exact tests were used for categorical variables. Characteristics of and adverse events related to sedation were analyzed, except in patients who received general anesthesia. *P* values <0.05 were accepted as statistically significant, except where otherwise specified. The post-hoc pairwise comparisons between groups for airway interventions (oral/nasal airway, LMA, intubation) used the standard residual method, as suggested by Beasley and Schumacker [7], and results were reported as Bonferroni-adjusted P values.

## Results

There were 897 pediatric patients who received sedation for MRI examination during the study period. Of those, 356 received propofol alone (Group P), and 541 received propofol with midazolam (Group PM). Four patients in Group P and four patients in Group PM received general anesthesia with placement of an endotracheal tube or laryngeal mask airway during the MRI scan.

The demographic data and MRI type are presented in Table 1. There were no differences in demographic data between the two groups. Airway interventions used during sedation are presented in Table 2. The frequency of all airway interventions was 25/356 (7.0%) in Group P and 15/541 (2.8%) in Group PM (difference in proportions: 4.2%; 95% CI: 1.4–7.6%; *p* = 0.002). The frequency of oral/nasal airway application was 14/356 (3.9%) in Group P and 10/541 (1.8%) in Group PM (difference in proportions: 2.1%; 95% CI: 0–4.9%; *p* = 0.058). The frequency of LMA insertion was 11/356 (3.1%) in Group P and 4/541 (0.7%) in Group PM (difference in proportions: 2.4%; 95% CI: 0.1–4.8%; *p* = 0.007). The frequency of intubation was 0/356 (0%) in Group P and 1/541 (0.2%) in Group PM (difference in proportions: 0%; 95% CI: −0.9-1.0%; *p* = 0.417). There were no adverse events requiring airway intervention except for additional airway devices use.

**Table 1:** The demographic data and MRI type

	Group P (*N* = 356)	Group PM (*N* = 541)
Age (yr)	6.1 ± 2.6	5.8 ± 2.4
Weight (kg)	21.9 ± 8.5	21.2 ± 8.0
Sex (male/female)	197/159 (55/45)	294/247 (54/46)
ASA class (I/II)	133/223 (38/62)	228/313 (42/58)
MRI type		
Abdomen	8 (2.2)	9 (1.7)
Brain	183 (51.4)	248 (45.8)
Extremity	9 (2.5)	14 (2.6)
Mediastinum & Lung	12 (3.4)	46 (8.5)
Neck	19 (5.3)	23 (4.3)
Orbit	31 (8.7)	58 (10.7)
Parotid gland	3 (0.8)	5 (0.9)
Pelvis	3 (0.8)	14 (2.6)
Sella	33 (9.3)	30 (5.5)
Spine	42 (11.8)	53 (9.8)
Temporal lobe	13 (3.7)	41 (7.6)

Data are mean ± SD or number (%)

*ASA* American Society of Anesthesiology, *MRI* magnetic resonance imaging

**Table 2:** Airway intervention

	Group P (N = 356)	Group PM (N = 541)	P value
Overall airway interventions	25 (7.0)	15 (2.8)	0.005
Oral/nasal airway	14 (3.9)	10 (1.8)	0.058*
LMA	11 (3.1)	4 (0.7)	0.007*
Intubation	0 (0.0)	1 (0.2)	0.417*

Data are number (%). LMA, Laryngeal mask airway

*The Bonferroni-adjusted p value is set at 0.0083

The characteristics of sedation are presented Table 3. The anesthetic time was similar between the two groups. The mean (SD) propofol induction dose was higher in Group P compared to Group PM [2.4 (0.7) mg vs, 1.3 (0.5) mg; mean difference, 1.1 mg; 95% CI, 1.0–1.2; *p* < 0.001]. The mean (SD) infusion rate was higher in Group P compared to Group PM [161.3 (38.6) μg/min/kg vs. 116.2 (25.6) μg/min/kg; mean difference 45.0 μg/min/kg; 95% CI, 40.4 to 50.0; *p* < 0.001]. The mean (SD) propofol total dose was higher in Group P compared to Group PM [236.3 (102.4) mg vs. 18.7 (80.9) mg; mean difference, 55.5 mg; 95% CI, 42.8–68.2; p < 0.001]. The mean (SD) time to awake was longer in Group PM compared to Group P [21.2 (5.6) minutes vs. 23.0 (7.1) minutes; mean difference, 1.8 min; 95% CI, 0.9–2.9; p < 0.001]. The mean (SD) time to discharge was longer in Group PM compared to Group P [34.5 (6.9) minutes vs. 38.6 (9.4) minutes; mean difference, 4.0 min; 95% CI, 3.0–5.1; p < 0.001].

**Table 3:** The characteristics of sedation

	Group P (*N* = 352)	Group PM (*N* = 537)	P value
Anesthetic time (min)	39.7 ± 12.5	39.4 ± 11.1	0.871
Propofol induction dose (mg/kg)	2.4 ± 0.7	1.3 ± 0.5	<0.001
Infusion rate (μg/kg/min)	161.3 ± 38.6	116.2 ± 25.6	<0.001
Total propofol dose (mg)	236.3 ± 102.4	180.7 ± 80.9	<0.001
Time to awake (min)	21.2 ± 5.6	23.0 ± 7.1	<0.001
Time to discharge time (min)	34.5 ± 6.9	38.6 ± 9.4	<0.001

Data are mean ± SD. The data analyzed except eight patients who received general anesthesia

Adverse events during sedation are presented in Table 4. The frequency of movement, bradycardia, and hypotension during imaging was similar between the two groups. There were two patients with postoperative agitation in Group PM and one patient with desaturation in Group P. There were no serious events including aspiration, increased level of care, cardiac arrest and death during the MRI scan.

**Table 4:** Sedation-related adverse events

	Group P (*N* = 352)	Group PM (*N* = 537)	P value
Movement	7 (2.0)	13 (2.4)	0.818
Bradycardia	12 (3.4)	15 (2.8)	0.690
Hypotension	0 (0.0)	2 (0.4)	0.651
Desaturation (SpO_2_ < 90)	1 (0.3)	0 (0.0)	0.369
Postoperative agitation	0 (0.0)	2 (0.4)	0.521

Data are number (%). The data analyzed except eight patients who received general anesthesia

## Discussion

Propofol for pediatric MRI sedation increases the incidence of serious adverse events [2, 8]. In our study, the use of propofol with midazolam for pediatric MRI sedation performed by a pediatric anesthesiologist reduced the frequency of airway obstruction, but slightly increased the recovery profile.

Various intravenous sedative agents, such as propofol, midazolam, and dexmedetomidine, have been used for pediatric procedural sedation [2, 3, 9–11]. The use of propofol alone tends to increase the incidence of sedation-related serious adverse events, because it has dose-dependent response to upper airway collapse by inhibition of airway dilator muscle and of upper airway reflexes [2, 5, 8]. Therefore, concomitant administration of propofol with other sedative agents, such as midazolam, ketamine and dexmedetomidine, have been evaluated for pediatric procedural sedation [2, 4, 10]. Among various intravenous sedative agents for pediatric procedural sedation, propofol and midazolam have been preferred over others because of their high potency, short half-lives, and low potential of adverse effects [10]. In addition, concomitant administration of propofol with midazolam brings drug synergy effect on sedation and contributes to decreased risk of having adverse events [4, 6]. Therefore, by using midazolam and propofol combination regimen, it could provide short induction time, fast recovery, stable cardiorespiratory conditions, and rarely requires additional sedation, and therefore is safe and adequate for pediatric MRI sedation [6, 12]. However, midazolam has longer induction and recovery times than propofol, these disadvantages are less severe than those seen with dexmedetomidine [10, 11]. In our study, the recovery profile was prolonged in patients administered propofol in combination with midazolam. The time to awake and time to discharge in Group PM were 1.7 min and 4 min longer, respectively, compared to Group P. However, these time differences are not clinically relevant. These results might be attributed to the use of a small dose of midazolam. Therefore, we concluded that concomitant administration of a small dose of midazolam and propofol did not affect the recovery profile.

The frequency of airway interventions increased in Group P compared to Group PM. Among them, the frequency of use of LMAs was higher in Group P. We considered this to be due to the higher total and induction doses of propofol used in Group P compared with the doses used in Group PM. Because of the need for deep sedation in pediatric MRI examination, the frequency of use of LMA is typically high. However, the use of LMAs for pediatric MRI sedation patients during emergence from anesthesia has been reported to be associated with more airway-related problems than propofol sedation alone [13]. Therefore, attention should be paid to airway irritation, such as the presence of coughs, hiccups, or laryngospasms, when using LMAs. In Group PM, there was one patient who needed intubation. In this patient, intubation was performed due to interference with the brain MRI coil when using an LMA. Therefore, the possibility of intubation should be considered when airway obstruction occurs during brain MRI.

The auditory signal amplitude has been reported to decrease when sedation is achieved with midazolam and dexmedetomidine, but not with propofol [13]. These effects are also present with very low doses of midazolam and dexmedetomidine [13]. The high amount of noise generated by MRI equipment can cause movement during sedation. In our study, there was no difference in movement during MRI sedation between Group P and Group PM. When movement during MRI sedation occurred, the propofol infusion rate was increased or a bolus of propofol was administrated. In most cases, the movement disappeared, but a high dose of propofol was administered more frequently in patients in Group P compared to those in Group PM. In addition, administration of propofol was not effective in some cases. In these cases, movement disappeared with the administration of a small dose of midazolam. Therefore, in pediatric MRI sedation, the administration of a small dose of midazolam might be more effective than propofol when movement occurs.

In Group PM, postoperative agitation occurred in two patients. The cause of postoperative agitation can vary, but one possibility is that it occurs as a paradoxical reaction to midazolam, given that it only occurred in patients in Group PM. Paradoxical reactions to midazolam can be reversed with flumazenil [14]. As the symptoms were not severe, we monitored carefully and recovered completely without any complications. Paradoxical reactions to midazolam are more frequent in patients under 3 years of age and in patients given high doses of midazolam [15]. However, in our study, the incidence of paradoxical reactions was low because all patients were over 3 years old and were given a low dose of midazolam.

Our study has the following limitations. First, this is a single center, retrospective study. Thus, we cannot establish a causal relation between the administration of midazolam during pediatric MRI sedation and reduced the frequency of airway complications. In particular, one of the two drugs was selected at the discretion of anesthesiologists, thus, there was the risk of selection bias. Nonetheless, selection bias may have not significantly affected the results of the current study because other perioperative managements are standardized including airway intervention and the dose of sedative agents except for difference in sedation drugs regimen. Second, there was no record of the severity of airway obstruction or airway repositioning, so we were only able to compare the frequency of airway intervention. However, we performed optimal airway positioning before performing MRI in all patients.

## Conclusion

The administration of a small dose of midazolam during pediatric MRI sedation using propofol can reduce the frequency of airway complications without prolonging the clinically significant recovery profile.

## Abbreviations

ASA: American Society of Anesthesiology
LMA: laryngeal mask airway
MRI: magnetic resonance imaging
